# Pathogenic Variants in PET Complex Encoding Genes *TDRD12* and *EXD1* Impair piRNA Biogenesis and Cause Male Infertility

**DOI:** 10.1155/humu/7629729

**Published:** 2026-08-21

**Authors:** Beibei Zhang, Ao Ma, Hongyi Wang, Chong Xie, Shengwei Ke, Ziqi Yu, Haoran Xu, Zhipeng Xu, Zhiran Li, Xun Wang, Lv Yao, Fuxing Zhang, Lingying Jiang, Xiaozhi Zhao, Fei Sun

**Affiliations:** ^1^ Department of Urology and Andrology, Sir Run Run Shaw Hospital, Zhejiang University School of Medicine, Hangzhou, Zhejiang, China, zju.edu.cn; ^2^ Department of Andrology, Nanjing Drum Tower Hospital, The Affiliated Hospital of Nanjing University Medical School, Nanjing, Jiangsu, China, nju.edu.cn; ^3^ Assisted Reproduction Unit, Department of Obstetrics and Gynecology, Sir Run Run Shaw Hospital, Zhejiang University School of Medicine, Hangzhou, Zhejiang, China, zju.edu.cn

**Keywords:** EXD1, male infertility, meiosis, PET complex, piRNA, TDRD12

## Abstract

**Background:**

Nonobstructive azoospermia (NOA), the most severe form of male infertility, has approximately 30% of its cases attributed to genetic factors. However, current genetic research only explains a small fraction of NOA cases.

**Methods:**

Here, 156 infertile Chinese patients diagnosed with idiopathic male infertility were recruited. Whole exome sequencing and subsequent bioinformatic analysis were performed to find candidate pathogenic variants. Sanger sequencing was used to validate the candidate variants. Quantitative reverse‐transcript PCR (RT‐qPCR) was performed to detect the mRNA expression of *TDRD12* and related piRNA genes. Hematoxylin and eosin staining and immunofluorescence staining were performed on testicular sections obtained from patients′ biopsies. Intracytoplasmic sperm injection (ICSI) was applied for assisted fertilization. Additionally, small RNA sequencing was performed to identify the abnormalities of piRNA biogenesis.

**Results:**

We identified eight variants in PET (PIWI‐EXD1‐TDRD12) complex encoding genes *TDRD12* and *EXD1* from three NOA patients and one oligoasthenozoospermia patient. These variants, which were rare or absent in public human databases, were predicted to be deleterious. Notably, *TDRD12* mRNA was absent in three NOA patients. Testicular histopathological analyses revealed that spermatogenesis was arrested at zygotene stage in patients carrying biallelic *TDRD12* variants. Further immunofluorescence staining of L1ORF1p and small RNA sequencing revealed disruptions in piRNA biogenesis and subsequent derepression of retrotransposons. Further assisted reproduction outcomes showed that one of the individuals conceived successfully after ICSI.

**Conclusion:**

Overall, this study significantly expands the mutational phenotype of PET complex‐encoding genes and significantly advances our understanding of spermatogenic failure and male infertility. Our findings provide direct clinical and functional evidence that the function of the PET complex and piRNA biogenesis is highly conserved between humans and mice.

## 1. Introduction

Nonobstructive azoospermia (NOA), the most severe form of male infertility resulting from impaired sperm production, affects approximately 1% of all men [1]. Genetic factors, including abnormal karyotypes, Y‐chromosome microdeletions, and gene variants, account for ~30% of NOA cases [2]. Recent advances in next‐generation sequencing technologies have significantly accelerated the identification of single‐gene variants associated with NOA. To date, whole exome sequencing (WES) analyses have revealed variants in genes such as *STAG3* (MIM: 608489), *KCTD19* (MIM: 619943), *REDIC1* (MIM: 620495), and *SIX6OS1* (MIM: 617307) [3–8], as well as in several piRNA pathway genes, including *PIWIL1* (MIM: 605571), *PNLDC1* (MIM: 619529), *TDRD1* (MIM: 605796), and *TDRD9* (MIM: 617963) [9–12], all of which are implicated in spermatogenesis and male infertility. Despite these discoveries, current genetic findings explain only a small fraction of NOA cases [13], leaving the etiology and pathology of the majority of infertile individuals still largely unknown.

P‐element‐induced wimpy testis (PIWI)–interacting RNAs (piRNAs) are a class of small noncoding RNAs that play a critical role in spermatogenesis by silencing transposon activity during the epigenome reprogramming of fetal germ cells [14]. The biogenesis of piRNAs is a multistep process: piRNA precursors are first transcribed from piRNA clusters, then cleaved by endonucleases, and subsequently bound by PIWI proteins to form pre‐piRNAs. These pre‐piRNAs are further trimmed by 3 ^′^–5 ^′^ exonucleases to their mature length, which is determined by the region protected by the bound PIWI protein [14]. The PET (PIWI‐EXD1 [exonuclease domain‐containing 1]‐TDRD12) complex has been reported to play a pivotal role in MILI slicing [15], a process essential for the biogenesis of most piRNAs associated with MIWI2 [16]. TDRD12, a Tudor domain‐containing protein, is crucial for spermatogenesis, as its deficiency leads to zygotene arrest, widespread derepression of LINE1 elements, and impaired production of secondary piRNAs that interact with PIWIL4 in *Tdrd12* knockout mice [17]. Recent studies have identified compound heterozygous variants in *TDRD12* as a cause of spermatogenic failure in an NOA patient, underscoring the conserved role of TDRD12 in humans [18]. EXD1, an interaction partner of TDRD12 within the PET complex, functions as an RNA adaptor rather than an active nuclease [15]. Loss of EXD1 only modestly reduces MIWI2 piRNA levels and does not affect fertility in mice [15], its knockout in the *Tdrd12* ^+/−^ background (*Exd1* ^−/−^; *Tdrd12* ^+/−^) results in a dramatic reduction in MIWI2 piRNAs, leading to male infertility [19]. These findings highlight the TDRD12‐EXD1 complex as an indispensable component of the secondary piRNA biogenesis pathway.

In this study, we recruited 156 unrelated male infertility patients, comprising 108 idiopathic NOA patients and 48 oligo/astheno/teratozoospermia (OAT) patients, from multiple centers across China. WES was performed to identify potential pathogenic variants responsible for male infertility. Notably, biallelic variants in *TDRD12* were identified in 3 out of 108 genetically unexplained NOA patients. The absence of *TDRD12* mRNA in the testes of these patients suggested the degradation of mutant transcripts. Histopathological analyses further confirmed zygotene arrest in spermatogenesis and the derepression of retrotransposons. In addition, compound heterozygous *EXD1* variants and a heterozygous *TDRD12* missense variant were identified in 1 out of 48 oligoasthenozoospermia patients, aligning with previous functional studies on the PET complex in both humans and mice [15, 17–19]. Small RNA sequencing revealed disruptions in piRNA biogenesis, including altered piRNA patterns and lengths, in the NOA patients. Collectively, our findings demonstrate that variants in the PET complex‐encoding genes *TDRD12* and *EXD1* contribute to male infertility by impairing piRNA biogenesis.

## 2. Methods

### 2.1. Study Participants

A total of 156 infertile Chinese men, including 108 diagnosed with NOA and 48 diagnosed with OAT, were recruited in this study. Peripheral blood samples were obtained from all the patients. Karyotypes analysis, serum reproductive hormones, palpation of the scrotal contents, scrotal ultrasound, and/or testicular histology were performed in the local clinical laboratories. Routine semen analysis was performed for all the patients according to the WHO guidelines [20]. Parental peripheral blood samples were unavailable. Written informed consent forms from all participants were obtained at the beginning of the study according to the ethics committee of Sir Run Run Saw Hospital, Zhejiang University of Medicine (approval number 2022‐726‐01).

### 2.2. WES and Variants Filtering

WES was performed for all the recruited patients. In brief, total genomic DNA was extracted from the peripheral blood samples using the QIAamp DNA Blood Mini Kit (QIAGEN, 51106). Whole‐exome capture was conducted using the AlExome Enrichment Kit V1 (iGeneTech), followed by sequencing on an Illumina NovaSeq platform (Illumina). Reads were aligned to the human reference genome (GRCh37/hg19) using Burrows–Wheeler Aligner [21].

Variants calling was performed using GATK4 [22] to identify single‐nucleotide variants (SNVs) and small insertions/deletions (indels), followed by ANNOVAR annotation [23] against clinical and population databases, including gnomAD, ExAC, and 1000 Genomes. Variants were classified according to ACMG guidelines [24]. Initial filtration retained protein‐altering variants with MAF < 0.001 in public databases, predicted pathogenicity (REVEL ≥ 0.65, phastCons ≥ 0.9), and testis‐specific expression (TPM ≥ 10). Following exclusion of known NOA/OAT‐associated genes, the remaining 167 candidate genes underwent manual curation. Priority was given to genes implicated in spermatogenesis (Figure S1). Of note, for Patient OAT‐0190, a *TDRD12* missense variant with MAF > 0.001 was manually retained because of the identification of rare compound heterozygous *EXD1* variants and the hypothesis of digenic inheritance. Final candidate variants were validated by Sanger sequencing. Sequences of primers used for Sanger sequencing are listed in Table S1.

### 2.3. Quantitative Reverse‐Transcript PCR (RT‐qPCR)

Total RNAs were extracted from either testicular sperm aspiration remnants using RNAiso Plus reagents (TaKaRa, 9109) or from paraffin‐embedded testicular sections using the RNeasy FFPE Kit (Qiagen, 73504), following manufacturer′s protocol. cDNA synthesis was performed with PrimeScript RT reagent kit (TaKaRa, RR047A). Quantitative RT‐qPCR was performed using TB Green Premix Ex Taq (Takara, RR420A) on a CFX Real‐Time PCR System (BIO‐RAD, 1855204). The relative expression was normalized to *ACTB*. Primers sequences are listed in Table S2.

### 2.4. Hematoxylin and Eosin (H&E) Staining

Testicular biopsies were collected and fixed in Bouin′s or formalin solution, paraffin‐embedded, and sectioned at 5 *μ*m thickness. H&E staining was performed as we previously reported [6]. The images were captured using an Olympus BX43F microscope equipped with an SC180 camera and processed with cellSens Entry software (OLYMPUS, Japan).

### 2.5. Immunofluorescence Staining of Testicular Sections

Testicular tissues were fixed in formalin, paraffin‐embedded, and sectioned at 5 *μ*m. After permeabilization with 0.2% Triton X‐100 in PBS, sections were blocked with DBT solution (3% BSA and 10% normal donkey serum in tris‐buffered saline) for 30 min, followed by incubation with primary antibodies at 4°C overnight. After three washes in PBS containing 0.1% Triton X‐100 (PBST), secondary antibodies were applied for 1 h at 37°C. Subsequently, the slides were washed three times in PBST, air dried, counterstained with Hoechst 33342 (Invitrogen, H21492), and mounted with VECTASHIELD (Vector Laboratories, H‐1000). Images were captured using a ZEISS LSM 980 with Airyscan 2 and processed using ZEISS ZEN software (Germany). Antibodies used were listed in Table S3.

### 2.6. Small RNA Sequencing

The human testicular RNA was extracted from paraffin‐embedded sections of individuals NOA‐0056 and A15626, and three obstructive azoospermia (OA) patients who have given birth to a child, using the Total RNA Purification Kit (LC Sciences, United States) according to the manufacturer′s protocol. The total RNA quantity and purity were analysis by Bioanalyzer 2100 and RNA 6000 Nano LabChip Kit (Agilent, California, United States), showing RIN number > 7.0. Small RNA library was prepared from 1 *μ*g of total RNA using TruSeq Small RNA Sample Prep Kits (Illumina, San Diego, USA), followed by 50 bp single‐end sequencing on an Illumina Hiseq2500 platform at LC‐BIO (Hangzhou, United States), according to the vendor′s recommended protocol.

### 2.7. Differential Expression Analysis of piRNAs

Raw sequencing reads were trimmed and filtered for quality, then aligned to the human reference genome (GRCh37/hg19) using the piRNA pipeline. piRNA expression levels were quantified as reads per million (RPM). Differential expression analysis between NOA patients (*n* = 2) and OA controls (*n* = 3) was performed using DESeq2 (Version 1.32.0) with a negative binomial model. Genes (piRNAs) with an adjusted *p* < 0.05 (Benjamini–Hochberg correction for multiple testing) and an absolute log_2_ (fold change) > 1 were considered significantly differentially expressed. Target genes of significantly downregulated piRNAs (adjusted *p* < 0.05, |2*F* *C*| > 1) were predicted using miRanda (Version 3.3a) with default parameters. Gene Ontology (GO) enrichment and Kyoto Encyclopedia of Genes and Genomes (KEGG) pathway enrichment analyses were performed using clusterProfiler (Version 4.0.5) in R. Enriched terms with an adjusted *p* < 0.05 (Benjamini–Hochberg correction) were considered statistically significant.

### 2.8. Expression Constructs and Cell Culture

Codon‐optimized coding sequences of human EXD1 (wild‐type [WT] and two mutants, V45M and L303X) were synthesized and cloned into the pEGFP‐N1 vector (Tsingke) to generate N‐terminal HA‐tagged and C‐terminal mCherry‐tagged fusion proteins. HEK293T cells (ATCC, CRL‐3216) were cultured in DMEM (Gibco) supplemented with 10% (v/v) fetal bovine serum (Gibco) and 1% penicillin‐streptomycin (Gibco) at 37°C in a humidified atmosphere containing 5% CO_2_. Cells were passaged two to three times after thawing and seeded to reach 70%–80% confluency prior to transfection. Transient transfections were performed using PEI MAX (Polysciences, 24765) following the manufacturer′s protocol. All plasmid DNA used was endotoxin‐free. Transfected cells were cultured for 48 h before further analysis.

## 3. Results

### 3.1. *TDRD12* and *EXD1* Variants Identified in Chinese Infertile Individuals

To investigate the genetic causes of human infertility, we recruited 156 Chinese individuals presenting with idiopathic infertility from multiple centers. The patients reported in this study were recruited from distinct geographic regions in China, NOA‐0056 and OAT‐0190 originated from Zhejiang province, NOA‐0064 from Shandong province, and A15626 from Jiangsu province. All patients exhibited normal male phenotypes according to the consultation and ultrasonography examinations except the slightly reduced testicular size in Patients NOA‐0056, NOA‐0064, and OAT‐0190 (Table 1), consistent with the clinical presentation of NOA or OAT. Karyotype analysis revealed a normal 46, XY‐chromosome configuration in all patients, with no detectable Y‐chromosome microdeletions. Routine semen analysis demonstrated normal semen volume but a complete absence of sperm in ejaculate pellet after high‐speed centrifugation in NOA‐0056, NOA‐0064, and A15626. In contrast, Patient OAT‐0190 exhibited severely reduced sperm concentration and a complete lack of motile sperm (Table 1). Hormonal profiling indicated normal reproductive hormone levels in Patients NOA‐0056 and OAT‐0190, with the exception of low prolactin levels. Patient NOA‐0064 displayed high follicle‐stimulating hormone and testosterone levels alongside reduced prolactin levels. Hence, Patients NOA‐0056, NOA‐0064, and A15626 were diagnosed with NOA, whereas OAT‐0190 was diagnosed with oligoasthenozoospermia. Notably, OAT‐0190 has received intracytoplasmic sperm injection (ICSI) treatment, resulting in a successful clinical pregnancy for his couple (Table 2), highlighting a potentially valuable therapeutic approach for similar cases.

**Table 1:** Clinical characteristics of patients carrying *TDRD12* and/or *EXD1* variants.

	Reference values	NOA‐0056	NOA‐0064	A15626	OAT‐0190
Genetics					
*TDRD12* mutation	—	Homozygous missense	Compound heterozygous stopgain	Compound heterozygous frameshift and stopgain	Heterozygous missense
*EXD1* mutation	—	NA	NA	NA	Compound heterozygous missense and stopgain
Karyotype	—	46, XY	46, XY	46, XY	46, XY
Y‐chromosome microdeletion	—	No	No	No	No
Patient history					
Region	—	Zhejiang, China	Shandong, China	Jiangsu, China	Zhejiang, China
Age (years)^a^	—	26	31	30	32
Years of marriage^a^	—	Premarital check‐up	2	4	6
Height/weight (cm/kg)	—	174/75	173/114	172/66	170/80
Phenotype					
Pubertal development	—	Normal	Normal	Normal	Normal
Congenital maldescended testicles	—	No	No	No	No
Testis size (mL)	> 12.5	R:9.9; L:9.3	R:12.6; L:11.8	Unknown	R:11.5; L:12.9
Epididymis	—	Bilateral present;	Bilateral present;	Unknown	Bilateral present;
		Not enlarged	Not enlarged		Not enlarged
Results of transrectal ultrasonography	—	Prostate normal;	Prostate normal;	Unknown	Prostate normal;
		Vesicula seminalis normal	Vesicula seminalis normal		Vesicula seminalis normal
Varicocele	—	No	No	No	No
Hypospadias	—	No	No	No	No
Clinical diagnosis	—	NOA	NOA	NOA	Oligoasthenozoospermia
Semen analysis^b^					
Semen volume (mL)	> 1.5	2.2	2.8	3.2	5.08 ± 0.56
Semen pH	7.2–7.8	7.2	7.0	7.4	6.97 ± 0.43
Sperm concentration (10^6^/mL)	> 15	0	0	0	1.63 ± 0.77
Spermatozoa in ejaculate pellet	—	None	None	None	NA
Motile sperm (%)	> 40	NA	NA	NA	0
Progressively motile sperm (%)	> 32	NA	NA	NA	0
Hormone analysis^c^					
FSH (IU/L)	1.27–19.26	15.1	19.92	Unknown	3.42
LH (IU/L)	1.24–8.62	5.47	4.99	Unknown	2.67
Estradiol (pg/mL)	< 38.95	19.2	9.96	Unknown	27.56
Progesterone (*μ*g/L)	0.14–2.06	0.74	0.6	Unknown	Unknown
Testosterone (*μ*g/L)	1.75–7.81	7.37	14.56	Unknown	2.38
Prolactin (*μ*g/L)	2.64–13.13	2.26	1.97	Unknown	12.65
Testicular‐biopsy sample					
Fixative	—	Bouin’s and formalin fixative	Bouin’s and formalin fixative	Formalin fixative	NA
Leydig cell hyperplasia	—	No	No	No	NA
Spermatogenesis	—	Homogenous pattern with spermatogenic arrest	Homogenous pattern with spermatogenic arrest	Homogenous pattern with spermatogenic arrest	NA
RNA recovered	—	Yes, from remnants after microTESE	Yes, from remnants after microTESE	No	NA
Treatment					
TESA or microTESE	—	No sperm identified after microTESE	No sperm identified after microTESE	No sperm identified after TESA	NA
Treatment outcome	—	No pregnancy	No pregnancy	No pregnancy	NA

*Note:* For patients NOA‐0056, NOA‐0064, and A15626, one semen analysis was performed. For patient OAT‐0190, nine semen analyses were performed. Data are presented as mean ± SEM.

^a^The current age.

^b^Reference values were published by the World Health Organization (WHO) in 2021.

^c^Reference values were suggested by local clinical laboratory.

Abbreviations: FSH, follicle‐stimulating hormone; L, left; LH, luteinizing hormone; microTESE, microsurgical testicular sperm extraction; NA, not available; NOA, nonobstructive azoospermia; R, right; TESA, testicular sperm aspiration.

**Table 2:** Clinical treatment and outcomes of the patient OAT‐0190.

Fertilization method	ICSI
Female age (years)^a^	29
Number of cycles	2
Number of oocytes injected	15
Number of fertilized oocytes	10
Number of cleavage embryos	9
Number of 8‐cells	1
Number of blastocysts	0
Number of transfer cycles	2
Number of embryos transferred per cycle	2
Treatment outcome	Clinical pregnancy

^a^The current age.

Abbreviation: ICSI, intracytoplasmic sperm injection.

**Figure 1:**
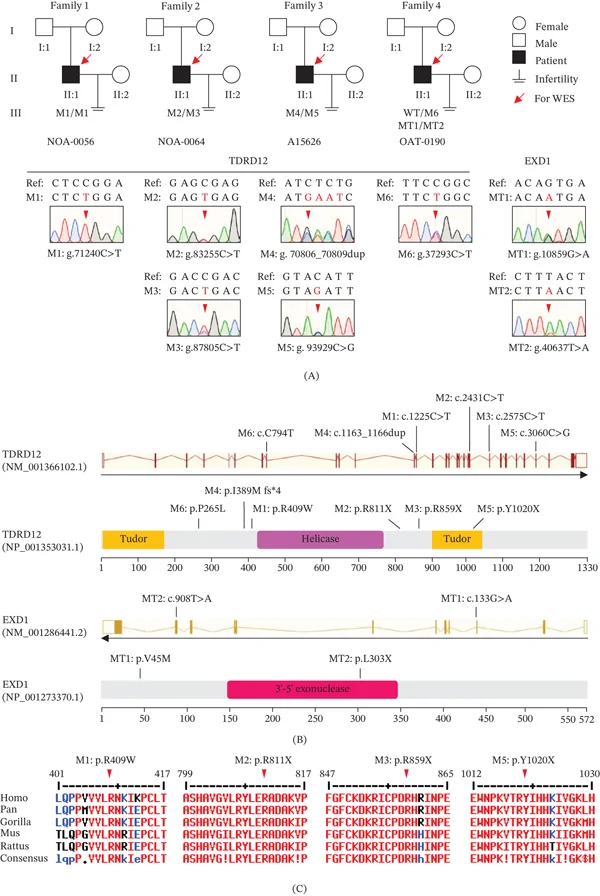
Biallelic *TDRD12* and *EXD1* variants identified in four Chinese infertile individuals. (A) Pedigrees of the families. Squares and circles denote male and female members, respectively. Solid symbols indicate the affected males, open symbols denote unaffected members, and the equal sign indicates infertility. Arrows point to the three individuals for which whole‐exome sequencing (WES) was performed. Sanger sequencing chromatograms of the *TDRD12* and *EXD1* variants in genomic DNA (NC_000019.10 and NC_000015.10, respectively) from all affected individuals are shown under the pedigrees. The nucleotides affected are written in red. The arrowheads indicate the variant sites. (B) Schematic representation of the variant positions in TDRD12 and EXD1 at the transcript level (NM_001366102.1 and NM_001286441.2, respectively) and protein level (NP_001353031.1 and NP_001273370.1, respectively). (C) Sequence alignment shows conservation of the affected amino acids in TDRD12, indicated by red arrowheads, across different organisms.

To identify the potential pathogenic genetic variants, WES was conducted on genomic DNA extracted from all participants. Through a stringent variant filtration process, eight variants in genes encoding the PET complex, comprising six *TDRD12* variants and two *EXD1* variants, were identified in four unrelated patients (Figure 1A). Specifically, Patient NOA‐0056 (Family 1, II:1, 26 years old) harbored a homozygous *TDRD12* missense variant (M1: c.1225C>T, p.R409W), whereas Patient NOA‐0064 (Family 2, II:1, 31 years old) carried compound heterozygous *TDRD12* nonsense variants (M2: c.2431C>T, p.R811X; M3: c.2575C>T, p.R859X). Patient A15626 (Family 3, II:1, 30 years old) exhibited compound heterozygous *TDRD12* variants, including a frameshift variant and a nonsense variant (M4: c.1163_1166dup, p.I389Mfs ^∗^4; M5: c.3060C>G, p.Y1020X). Additionally, Patient OAT‐0190 (Family 4, II:1, 32 years old) carried a heterozygous *TDRD12* missense variant (M6: c.C794T, p.P265L) and compound heterozygous *EXD1* variant (MT1: c.133G>A, p.V45M; MT2: c.908T>A, p.L303X) (Figure 1A,B). Although the *TDRD12* p.P265L variant has a relatively high minor allele frequency (0.0182 in gnomAD, exceeding our initial MAF threshold of 0.001), it was reincluded for further analysis because we hypothesized that a single TDRD12 missense variant might become pathogenic when co‐occurring with biallelic EXD1 loss‐of‐function, a digenic scenario supported by the *Exd1* ^−/−^; *Tdrd12* ^+/−^ mouse model [19]. All variants were validated by Sanger sequencing (Figure 1A).

These variants were either rare or absent in major public human genetics databases, including 1000 Genomes, ESP6500, ExAc, GnomAD, and GnomAD‐EAS (Table 3). With the exception of M1, M6, and MT1, all other variants introduced a premature stop codon, likely resulting in truncated or nonfunctional proteins (Figure 1B). The M1 and MT1 variants were predicted to be deleterious by in silico analysis via SIFT, PolyPhen‐2, and MutationTaster tools (Table 3). Furthermore, all variants except M4 exhibited high conservation across mammalian species (Figure 1C and Figure S2). Collectively, these findings strongly suggest that the eight variants are likely pathogenic and contribute to the infertility observed in these four patients.

**Table 3:** Biallelic *TDRD12* and *EXD1* variants identified in infertile men.

	NOA‐0056	NOA‐0064		A15626		OAT‐0190		
Gene	*TDRD12*	*TDRD12*		*TDRD12*		*TDRD12*	*EXD1*	
cDNA mutation	c.1225C>T	c.2431C>T	c.2575C>T	c.1163_1166dup	c.3060C>G	c.C794T	c.133G>A	c.908T>A
Protein alteration	p.R409W	p.R811X	p.R859X	p.I389M fs ^∗^4	p.Y1020X	p.P265L	p.V45M	p.L303X
Mutation type	Missense	Stopgain	Stopgain	Frameshift	Stopgain	Missense	Missense	Stopgain
Allele frequency								
1000 Genomes	0	0	0	0	0	0	0	0
ESP6500	0	0	0	0	0	0.012	0	0
ExAC	0	0	0	0	0	0.0049	0	0
gnomAD	0.00009204	0.00001314	0.00001972	0	0	0.0182	0	0
gnomAD‐EAS	0.0006	0	0	0	0	0.0212	0	0
Functional prediction								
SIFT	T	NA	NA	NA	NA	T	NA	T
PolyPhen‐2	B	NA	NA	NA	NA	B	P	NA
MutationTaster	D	D	D	NA	D	NA	D	D

*Note:* The accession number of *TDRD12* and *EXD1* are NM_001366102.1 and NM_001286441.2, respectively.

Abbreviations: B, benign; D, damaging; ExAC, Exome Aggregation Consortium; gnomAD, Genome Aggregation Database; gnomAD‐EAS, Genome Aggregation Database, East Asian population; NA, not available; P, polymorphism; T, tolerated.

### 3.2. Zygotene Arrest Were Observed in NOA Patients With *TDRD12* Loss‐of‐Function (LoF) Variants

**Figure 2:**
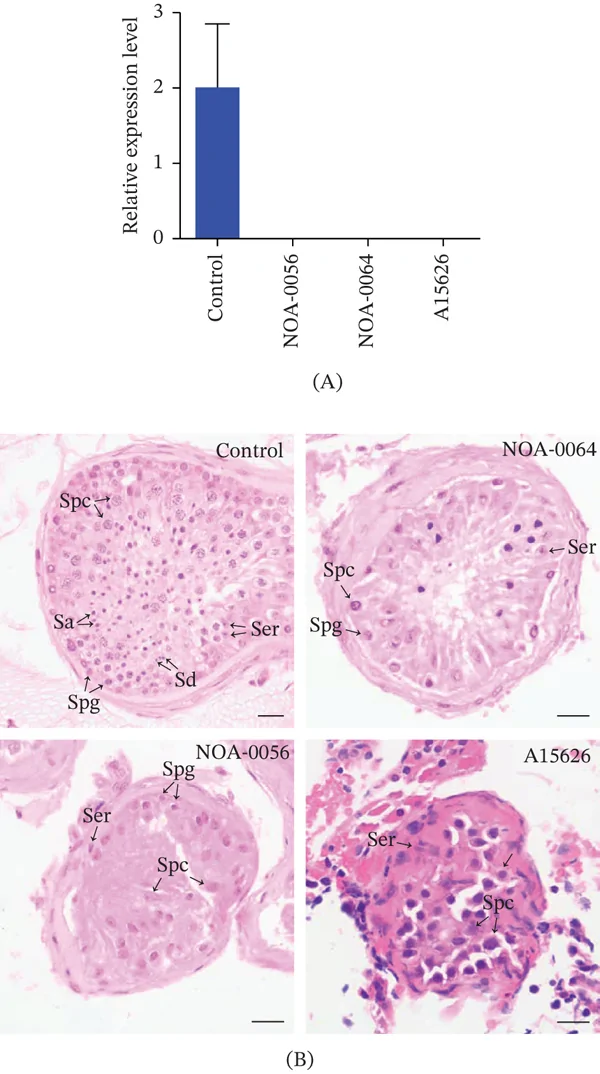
Patients with biallelic *TDRD12* variants displayed meiotic arrest. (A) Quantitative real‐time PCR analysis of *TDRD12* mRNA expression in testicular‐biopsy samples from all three affected individuals. Three pairs of primers downstream of the mutation site and two pairs of primers upstream of the mutation site were used and one replicate for each pair primers was used. *β*‐actin was used as an internal control. (B) Histological analyses of human testicular tissues by H&E staining. A fertile man with normal spermogram was used as the control. Abbreviations: Spg, spermatogonium; Spc, spermatocyte; Sa, Sd spermatid; Ser, Sertoli cell. Scale bars indicate 20 *μ*m.

Considering that the frameshift and nonsense variants introduce premature stop codons, potentially lead to mRNA decay or the production of truncated proteins, we first assess the relative expression of *TDRD12* mutant transcript using RT‐qPCR. Notably, *TDRD12* mRNA was undetectable in all three NOA patients (Figure 2A), suggesting that the mutant transcripts undergo nonsense‐mediated mRNA decay (NMD). Because *Tdrd12* knockout mice displayed meiotic arrest and male infertility [17], these five *TDRD12* variants are most likely pathogenic for infertility in all three NOA patients.

Testicular biopsies were obtained from the three NOA patients (NOA‐0056, NOA‐0064, and A15626), and a fertile man with normal spermatogenesis serving as the control. Histopathological examination of testicular biopsy samples from all three NOA patients showed that no spermatozoa were observed in both testicular sperm aspiration samples and microsurgical testicular sperm extraction samples. Seminiferous tubules from these NOA patients displayed a homogenous pattern with spermatogenic arrest (Table 2), further corroborating the diagnosis of NOA. H&E staining of testicular sections revealed a full spectrum spermatogenic cells, including spermatogonia, spermatocytes, and spermatozoa, in the seminiferous tubules of the control. In contrast, the seminiferous tubules of all three NOA patients contained spermatogonia and spermatocytes but lacked postmeiotic cells (Figure 2B). This result indicated that spermatogenesis was arrested at spermatocyte stage in the patients.

**Figure 3:**
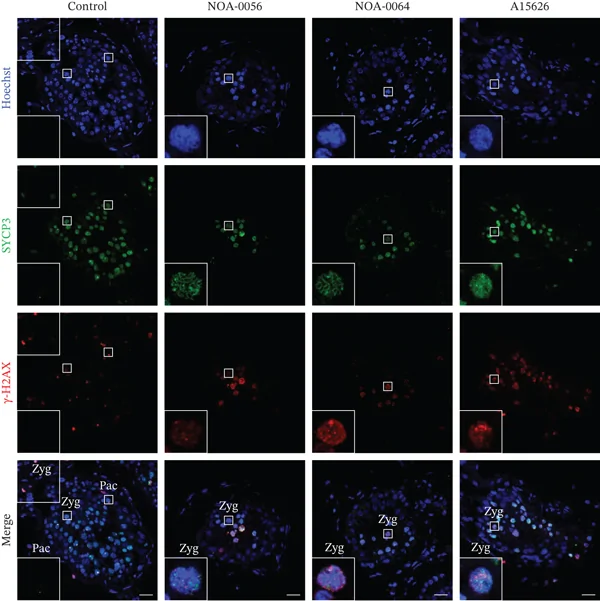
*TDRD12* variants led to meiotic arrest at zygotene stage in the male patients. Representative images of testicular sections from the control and all three affected individuals stained with antibodies against SYCP3 (green) and *γ*‐H2AX (red). The nuclei were stained with Hoechst 33342 (blue). Abbreviations: Zyg, zygotene; Pac, pachytene. Scale bars indicate 20 *μ*m.

To further explore the precise stage of meiotic arrest, immunofluorescence staining was performed on testis paraffin sections obtained from testicular biopsies. We utilized antibodies against SYCP3 (a lateral element of synaptonemal complexes) and *γ*‐H2AX (a marker of DNA double‐strand break) to evaluate meiosis progression. The results showed a reduced number of SYCP3‐positive cells in the patients′ seminiferous tubules. Importantly, although control samples exhibited SYCP3‐positive cells with *γ*‐H2AX‐marked XY bodies, characteristic of pachytene or diplotene spermatocytes, the SYCP3‐positive cells in patients displayed diffuse *γ*‐H2AX signals, indicating the absence of postzygotene spermatocytes (Figure 3). Additionally, staining with PNA (a marker of acrosome) revealed multiple PNA‐positive cells in the control seminiferous tubules, whereas no PNA‐positive cells were detected in the patients′ tubules, confirming the absence of spermatids (Figure S3). These findings align with the observations in *Tdrd12* knockout in testes [17], providing compelling evidence that the biallelic LoF variants in *TDRD12* identified in these NOA patients are indeed pathogenic, leading to meiotic arrest and male infertility.

### 3.3. piRNAs Biogenesis Defects in *TDRD12* Mutant Patients

**Figure 4:**
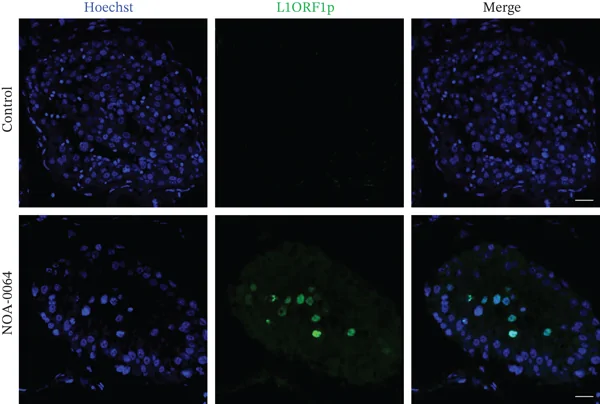
Retrotransposons are derepressed in the patients carrying biallelic *TDRD12* variants. Representative images of L1ORF1p (green) staining on testicular sections from the control and individual NOA‐0064. Nuclei were stained with Hoechst 33342 (blue). Scale bars indicate 20 *μ*m.

TDRD12 has been implicated in piRNA biogenesis and the repression of transposons. To investigate whether these processes were disrupted in patients with *TDRD12* variants, we assessed piRNA biogenesis and transposon expression. LINE1, a well‐characterized retrotransposon in the mouse genome, was examined by staining LINE1 ORF1p (L1ORF1p) on testicular sections from a normal control and the NOA patient. Although no signal of L1ORF1p was detected in the seminiferous tubules of the control, consistent with the effective transposon repression, robust L1ORF1p signals were observed in the spermatocytes of the NOA patient (Figure 4), indicating derepression of retrotransposons.

**Figure 5:**
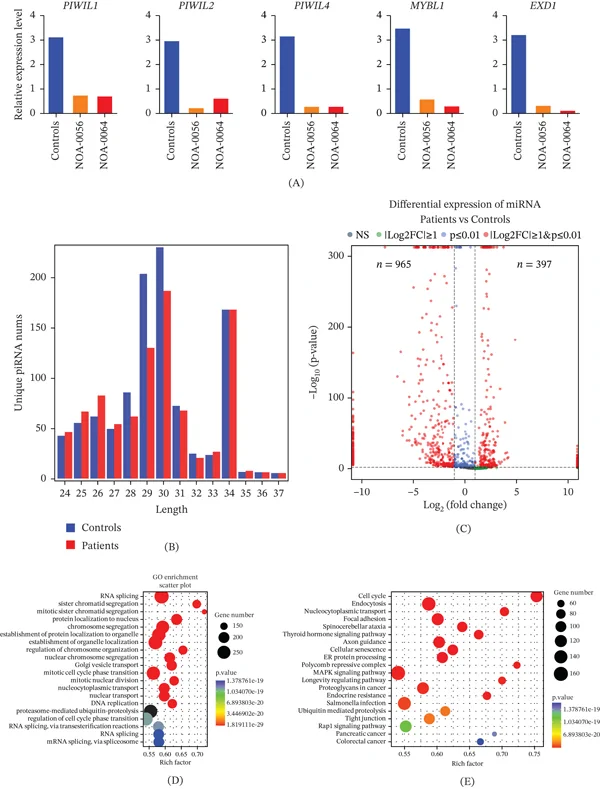
Expression of key piRNA‐processing enzymes at transcript level and small RNA‐sequencing. (A) Quantitative real‐time PCR analysis of piRNA‐processing associated genes *PIWIL1*, *PIWIL2*, *PIWIL4*, *MYBL1*, and *EXD1* in a control testicular‐biopsy sample with complete spermatogenesis and in samples from NOA‐0056 and NOA‐0064, who carried the biallelic *TDRD12* variants. *ACTB* was used as an internal control. (B) The read‐length distribution in piRNA libraries isolated from fixed testicular tissue sample from two patients NOA‐0056, A15626 and three controls who have given birth to a child. (C) Volcano plot showing differentially expressed piRNAs between NOA patients (*n* = 2) and OA controls (*n* = 3) based on DESeq2 analysis (adjusted *p* < 0.05, |log^2^ *f* *o* *l* *d* *c* *h* *a* *n* *g* *e*| > 1). Red dots indicate significantly upregulated piRNAs; blue dots indicate significantly downregulated piRNAs. (D) Gene Ontology (GO) enrichment and (E) Kyoto Encyclopedia of Genes and Genomes (KEGG) enrichment analyses of the gene to which the down‐regulated piRNA belongs in the patients compared with the controls. Only terms with adjusted *p* < 0.05 are shown.

To further evaluate the impact of *TDRD12* variants on piRNA biogenesis, we performed RT‐qPCR to measure the mRNA expression of piRNA‐related genes in Patients NOA‐0056 and NOA‐0064. The results demonstrated extreme reduction in the expression of key piRNA pathway genes, including *PIWIL1*, *PIWIL2*, *PIWIL4*, and *MYBL1*, in both NOA patients (Figure 5A). Additionally, the expression of *EXD1*, a piRNA‐related gene known to interact with TDRD12, was also reduced (Figure 5A). These results suggested that *TDRD12* variants seriously impair piRNA biogenesis in NOA patients.

Thus, small RNA sequencing was conducted to further elucidate the exact effects of *TDRD12* variants on piRNA biogenesis. The length distribution of piRNAs ranged from 24 to 37 bp, and the main different peaks were distributed at 29–30 and 34 bp (Figure 5B). Differential expression analysis using DESeq2 (adjusted *p* < 0.05, |log2*F* *C*| > 1) identified 1068 piRNAs with significantly reduced expression in NOA patients compared to controls and 475 piRNAs with increased expression (Figure 5C, volcano plot). The piRNA length distribution differed significantly between patients and controls (Kolmogorov–Smirnov test, *p* = 0.008), with distinct peaks at 29–30 and 34 nt in patients (Figure 5B). GO and KEGG enrichment analyses were performed with the downregulated piRNAs and indicated that the unique piRNAs identified in these patients were predominantly associated with RNA splicing pathway and chromatid segregation pathway, which are critically linked to spermatogenesis and male infertility (Figure 5D,E).

In summary, our findings demonstrate that *TDRD12* variants disrupt piRNA biogenesis in NOA patients, leading to aberrant piRNA expression patterns and the derepression of retrotransposons, thereby contributing to the pathogenesis of male infertility.

### 3.4. Functional Characterization of EXD1 Variants In Vitro

To functionally assess the two EXD1 variants (MT1: c.133G>A, p.V45M; MT2: c.908T>A, p.L303X) identified in Patient OAT‐0190, we constructed eukaryotic expression plasmids encoding HA‐tagged EXD1‐mCherry fusion proteins for WT, V45M, and L303X. Each construct was transfected into HEK293T cells. Western blot analysis of whole‐cell lysates showed that both mutant proteins (V45M and L303X) were expressed at substantially higher levels than WT EXD1, with equal GAPDH loading across all samples (Figure S4A). Subcellular localization was examined by confocal microscopy. WT EXD1 was predominantly localized in the cytoplasm. In contrast, both V45M and L303X mutants displayed not only cytoplasmic but also prominent nuclear localization (Figure S4B). Importantly, light microscopy observation prior to cell harvest revealed that cells transfected with the mutant constructs consistently exhibited visibly lower cell density compared to WT‐transfected cultures, suggesting that these EXD1 variants may affect cell proliferation or survival (Figure S4C). Collectively, these in vitro data demonstrate that both EXD1 missense and truncating variants alter protein expression, subcellular distribution, and cellular fitness, supporting their potential pathogenic role in the context of the coexisting TDRD12 p.P265L variant.

## 4. Discussion

In this study, we recruited 108 Chinese NOA patients and 48 Chinese OAT patients. Through genetic analysis, we identified eight variants in genes encoding the PET complex, including five *TDRD12* variants (one homozygous and two compound heterozygous) in three NOA patients, as well as compound heterozygous *EXD1* variants and a heterozygous *TDRD12* missense variant in one oligoasthenozoospermia patient. These findings underscore the critical role of the PET complex in human spermatogenesis and male fertility. RT‐qPCR analysis confirmed extremely reduced *TDRD12* mRNA expression in the testes of NOA patients. Furthermore, we observed that NOA patients carrying biallelic *TDRD12* variants exhibited abnormal piRNAs biogenesis and zygotene arrest during spermatogenesis, whereas the patient with biallelic *EXD1* variants and a *TDRD12* heterozygous missense variant displayed reduced sperm concentration and motility, consistent with previous functional studies of TDRD12 and EXD1 [15, 17–19]. Small RNA sequencing revealed disrupted piRNA biogenesis, particularly in piRNA expression patterns and length distributions, in NOA patients. Collectively, our study identifies novel variants in PET complex‐encoding genes that contribute to NOA and oligoasthenozoospermia by disrupting piRNA biogenesis.

Importantly, we considered that NOA‐0064 and A15626 carried compound heterozygous *TDRD12* variants without getting blood sample from their parents. According to the mRNA expression results of *TDRD12* in all three NOA patients, mRNA of *TDRD12* was totally lost or extremely reduced in these patients′ testes. There is no possibility that NOA‐0064 or A15626 carried heterozygous *TDRD12* variants with one strand expressing *TDRD12* normally. Thus, we concluded that NOA‐0064 and A15626 carried compound heterozygous *TDRD12* variants but not double variants in the same strand.

piRNAs are a class of small regulatory RNAs predominantly expressed in the male germline [25–27]. One of the main functions of piRNAs is to preserve the derepression of retrotransposons, which is often detected by the expression of LINE1 ORF1p, a classic retrotransposon. Unlike other small noncoding RNAs, piRNAs are generated through two distinct pathways: the primary processing pathway, which involves long precursor transcripts, and the secondary ping‐pong amplification pathway [14]. Initially, piRNA precursors are transcribed from piRNA clusters and subsequently cleaved by endonucleases, enabling their binding to PIWI proteins to form pre‐piRNAs. These pre‐piRNAs are then trimmed by 3 ^′^–5 ^′^ exonucleases to their mature length, determined by the region protected by the bound PIWI protein [14]. Given the role of TDRD12 and EXD1 in facilitating the binding of PIWI proteins to pre‐piRNAs, which influences the precise length of secondary piRNAs, we characterized the length distribution of piRNAs in *TDRD12* mutant patients and fertile controls. The length of secondary piRNAs ranged from 24 to 32 nucleotides; basically, the length of MILI‐interact piRNAs ranged from 26 to 28 nucleotides, the length of MIWI‐interact piRNAs ranged from 29 to 32 nucleotides, and the length of MIWI2‐interact piRNAs ranged from 26 to 31 nucleotides [14]. Notably, the most significant difference in unique piRNA length occurred at 28–30 nucleotides in the NOA patient, suggesting a pronounced effect on piRNAs interacted with all three classic PIWI proteins. Interestingly, the piRNA peak in 34 nucleotides is also different in NOA patient and control, indicating the potential difference in pre‐piRNA in NOA patient. This observation aligns with previous findings in *Tdrd12* knockout mice, which also highlighted the role of TDRD12 in MIWI2‐related piRNA biogenesis [17, 19].

In this study, we evaluated the mRNA expression of mutant *TDRD12*, identifying one homozygous missense variant and two compound stop‐gain variants. Interestingly, all biallelic *TDRD12* variants resulted in severely reduced *TDRD12* mRNA expression. Although NMD is commonly observed in patients with stop‐gain variants [28–30], the reduced mRNA expression caused by missense variants is rare [31]. In human spermatogenesis, several missense variants have been reported to significantly decrease mRNA expression, but the mechanisms remain unclear [32, 33]. Recently, several research have reported that even synonymous variants may alter mRNA expression due to the change of mRNA secondary structure or modification [34–37]. Because NMD is considered to rely on the ribosome stalling at stop codon [38], we suppose that the missense variant of *TDRD12* identified in this study may alter the secondary structure or modification of mRNA, leading to ribosome stalling and subsequent NMD.

The PET complex comprises PIWI proteins, TDRD12, and EXD1. TDRD12 is known to interact with MILI and is essential for the biogenesis of MIWI2‐associated piRNAs [17, 39]. Recently, *TDRD12* variants were identified in Han Chinese NOA patients, further implicating *TDRD12* as a pathogenic gene for NOA [18]. EXD1, which interacts with TDRD12, enhances MIWI2 piRNA biogenesis and derepresses LINE1 retrotransposons in mice [15]. Notably, although *Exd1* ^−/−^ mice are fertile, *Exd1* ^−/−^; *Tdrd12* ^+/−^ mice exhibited total infertility [19]. In this study, three NOA patients carried biallelic *TDRD12* variants, whereas Patient OAT‐0190 carried heterozygous *TDRD12* variant and biallelic *EXD1* variants, which contrasts with the normal fertility of *Exd1* ^−/−^ mice. We propose two nonexclusive explanations. First, the *TDRD12* p.P265L missense variant may exert a dominant‐negative effect, disrupting PET complex assembly beyond simple loss of function. Second, the combination of EXD1 LoF and TDRD12 p.P265L produces a synergistic effect that exceeds additivity, similar to *Exd1* ^−/−^; *Tdrd12^+/−^* mice, which are infertile [19]. The partial spermatogenic failure in OAT‐0190 (oligoasthenozoospermia, not azoospermia) suggests a dose‐dependent threshold effect. This case illustrates that individually tolerable variants can become pathogenic when co‐occurring in the same pathway.

Several limitations of this study should be acknowledged. First, the control group for small RNA sequencing consisted of three OA patients with histologically normal spermatogenesis, rather than truly fertile men. Although OA patients represent the best available control given the ethical constraints on obtaining testicular biopsies from healthy volunteers, we cannot exclude the possibility of secondary molecular changes induced by sperm outflow obstruction. Second, the small number of NOA patients (*n* = 2) and OA controls (*n* = 3) precluded formal statistical comparison of piRNA length distributions and limited the power of differential expression analysis; therefore, the observed differences are presented qualitatively and should be interpreted with caution. Third, due to the limited tissue availability and the patients′ refusal to provide additional samples, we were unable to perform *EXD1* expression analysis in the OAT patient or confirm the trans configuration of compound heterozygous variants by long‐range PCR. Future studies with larger sample sizes and functional validation will be necessary to fully establish the role of the PET complex in human spermatogenesis.

## 5. Conclusions

This study identifies eight pathogenic variants in PET complex‐encoding genes (five *TDRD12* variants in three NOA patients and two *EXD1* variants plus one *TDRD12* variant in one OAT patient) that lead to spermatogenic failure and male infertility. These findings provide compelling evidence that *TDRD12* and *EXD1* are crucial pathogenic genes for NOA and OAT, respectively, demonstrating the essential role of the PET complex in human spermatogenesis. This work significantly advances the understanding of genetic causes of male infertility and the molecular function of the PET complex in humans, offering valuable insights for clinical genetic diagnosis and counseling of affected patients.

## Author Contributions

B.Z., A.M., S.K. and H.W. performed the experiments, collected patient samples, interpreted the data, and drafted the manuscript. C.X., L.Y., F.Z., Z.X., Z.L., and X.W. recruited the patients. Z.Y. performed the sequencing data analysis. F.S., X.Z., L.J, and B.Z. conceived and supervised the study and gave insightful discussion and constructive comments on the manuscript. B.Z., A.M., H.W., and C.X. contributed equally to this work.

## Funding

This work was supported by the National Natural Science Foundation of China, 10.13039/501100001809, 82401865, 82371613, 92168112, 82070703; the Key Research and Development Program of Zhejiang Province, 10.13039/100022963, 2023C03035; the National Key Research and Development Program of China, 10.13039/501100012166, 2021YFC2700200; the Key Research and Development Program of Ningxia Hui Autonomous Region, 2021BEG02029; and the Jiangsu Funding Program for Excellent Postdoctoral Talent, 2024ZB030.

## Disclosure

All authors approved the final version of the manuscript.

## Conflicts of Interest

The authors declare no conflicts of interest.

## Supporting Information

Additional supporting information can be found online in the Supporting Information section.

## Supporting information


**Supporting Information 1** Figure S1: Whole‐exome sequencing (WES) data analysis pipeline. MAF, minor allele frequency. Figure S2: Sequence alignment shows conservation of the affected amino acids in EXD1, indicated by red arrowheads, across different organisms. Figure S3: Postmeiotic germ cells were absent in the patients carrying biallelic *TDRD12* variants. Representative images of PNA immunostaining (red) on testicular sections from all three affected individuals. Nuclei were counterstained with Hoechst 33342 (blue). A fertile man with normal spermogram was used as the control. Scale bars indicate 20 *μ*m. Figure S4: Functional characterization of EXD1 variants in HEK293T cells. (A) Western blot analysis of EXD1 and its variants expressed in HEK293T cells. GAPDH served as the loading control. (B) Representative confocal micrographs of HEK293T cells transiently transfected with plasmids HA‐EXD1‐mCherry (left), HA‐EXD1‐V45M‐mCherry (middle) and HA‐EXD1‐L303x‐mCherry (right), respectively. Nuclei were counterstained with Hoechst 33342. Scale bars indicate 5 *μ*m. (C) Representative differential interference contrast (DIC) images showing cell density at 48 h posttransfection. Nuclei were counterstained with Hoechst 33342. For A–C, three biological independent experiments were performed.

**Supporting Information 2** Table S1: Primers for human gDNA amplification and Sanger sequencing.

**Supporting Information 3** Table S2: Primers for human cDNA amplification.

**Supporting Information 4** Table S3: Antibodies used in this study.

**Supporting Information 5**  

## Data Availability

The data that supports the findings of this study are available in the supporting information of this article.
